# *Artemisia* Spp. Derivatives for COVID-19 Treatment: Anecdotal Use, Political Hype, Treatment Potential, Challenges, and Road Map to Randomized Clinical Trials

**DOI:** 10.4269/ajtmh.20-0820

**Published:** 2020-07-23

**Authors:** Paulin M. Kapepula, Jimmy K. Kabengele, Micheline Kingombe, Françoise Van Bambeke, Paul M. Tulkens, Antoine Sadiki Kishabongo, Eric Decloedt, Adam Zumla, Simon Tiberi, Fatima Suleman, Léon Tshilolo, Jean-Jacques Muyembe-TamFum, Alimuddin Zumla, Jean B. Nachega

**Affiliations:** 1 Faculty of Pharmaceutical Sciences, Centre d’Etudes des Substances Naturelles d’Origine Végétale (CESNOV), University of Kinshasa, Kinshasa, Democratic Republic of Congo;; 2 National Program for the Promotion of Traditional Medicine and Medicinal Plants (PNMT-PM), Ministry of Public Health, Kinshasa, Democratic Republic of Congo;; 3 Pharmacologie Cellulaire et Moléculaire, Louvain Drug Research Institute, Université Catholique de Louvain (UCLouvain), Brussels, Belgium;; 4 Department of Clinical Biology, Université Catholique de Bukavu (UCB), Bukavu, Democratic Republic of the Congo;; 5 Division of Clinical Pharmacology, Department of Medicine, Faculty of Medicine and Health Sciences, Stellenbosch University, Cape Town, South Africa;; 6 Barts and The London School of Medicine and Dentistry, Queen Mary University of London, London, United Kingdom;; 7 Pharmaceutical Sciences, University of KwaZulu Natal, Durban, South Africa;; 8 Unit of Sickle Cell Disease and Clinical Research, Monkole Hospital Center, Kinshasa, Democratic Republic of Congo;; 9 Le Centre de Formation et d’Appui Sanitaire (CEFA), Centre Hospitalier Monkole, Kinshasa, Democratic Republic of Congo;; 10 Department of Pediatrics, Official University of Mbuji-Mayi (UOM), Kinshasa, Democratic Republic of Congo;; 11 National Institute of Biomedical Research (INRB), Kinshasa, Democratic Republic of Congo;; 12 Department of Medical Microbiology and Virology, Faculty of Medicine, University of Kinshasa, Kinshasa, Democratic Republic of Congo;; 13 Division of Infection and Immunity, Department of Infection, Centre for Clinical Microbiology, University College London, London, United Kingdom;; 14 National Institute for Health Research Biomedical Research Centre, University College London, Hospitals, London, United Kingdom;; 15 Department of Medicine and Centre for Infectious Diseases, Faculty of Medicine and Health Sciences, Stellenbosch University, Cape Town, South Africa;; 16 Departments of Epidemiology and International Health, Johns Hopkins Bloomberg School of Public Health, Baltimore, Maryland;; 17 Department of Epidemiology, University of Pittsburgh Graduate School of Public Health, Pittsburgh, Pennsylvania;; 18 Departments of Epidemiology, Infectious Diseases, and Microbiology, University of Pittsburgh Graduate School of Public Health, Pittsburgh, Pennsylvania;; 19 Center for Global Health, University of Pittsburgh Graduate School of Public Health, Pittsburgh, Pennsylvania

## Abstract

The world is currently facing a novel COVID-19 pandemic caused by SARS-CoV-2 that, as of July 12, 2020, has caused a reported 12,322,395 cases and 556,335 deaths. To date, only two treatments, remdesivir and dexamethasone, have demonstrated clinical efficacy through randomized controlled trials (RCTs) in seriously ill patients. The search for new or repurposed drugs for treatment of COVID-19 continues. We have witnessed anecdotal use of herbal medicines, including *Artemisia* spp. extracts, in low-income countries, and exaggerated claims of their efficacies that are not evidence based, with subsequent political controversy. These events highlight the urgent need for further research on herbal compounds to evaluate efficacy through RCTs, and, when efficacious compounds are identified, to establish the active ingredients, develop formulations and dosing, and define pharmacokinetics, toxicology, and safety to enable drug development. Derivatives from the herb *Artemisia annua* have been used as traditional medicine over centuries for the treatment of fevers, malaria, and respiratory tract infections. We review the bioactive compounds, pharmacological and immunological effects, and traditional uses for *Artemisia* spp. derivatives, and discuss the challenges and controversies surrounding current efforts and the scientific road map to advance them to prevent or treat COVID-19.

## INTRODUCTION

The unprecedented COVID-19 pandemic caused by the novel zoonotic pathogen of humans, SARS-CoV-2, has, as of July 12, 2020, caused 556,335 deaths of 12,322,395 confirmed cases reported by the WHO.^1^ Although COVID-19 commonly presents as a severe respiratory tract illness, it causes multisystem disease, and deaths have been attributed to cytokine storm, acute respiratory distress syndrome (ARDS), and excessive aberrant immunological responses. Of several recent and ongoing treatment intervention trials, only two randomized controlled trials have to date demonstrated benefits of specific therapies. One study indicated that hospitalized COVID-19 patients who received remdesivir had a 31% faster time to recovery than those who received placebo.^2^ Another trial reported that dexamethasone reduced mortality by one-third in seriously ill patients requiring respiratory support.^3^ Of note, dexamethasone was previously shown to be effective in the treatment of ARDS.^4^ Among other drugs initially considered of promise, trials of chloroquine or hydroxycholoroquine plus lopinavir/ritonavir with or without azithromycin have shown no reduction in mortality in hospitalized patients.^5^

Despite limited success in finding effective treatments 7 months after the first appearance of SARS-CoV-2 as a new human pathogen, the desperate quest for new and repurposed drugs to reduce the morbidity and mortality of COVID-19 continues. The anecdotal use of *Artemisia* spp. extracts for COVID-19 treatment in low-income countries has led to exaggerated and unproven claims of its efficacy in the absence of a scientific basis or results from clinical trials. This highlights the urgent need for further research on herbal compounds to evaluate efficacy through controlled trials, and for efficacious compounds, to establish the active ingredients, develop formulations and dosing, and define pharmacokinetics, toxicology, and safety to enable drug development. We discuss the bioactive compounds, pharmacological and immunological effects, and traditional uses for *Artemisia* spp. derivatives, and discuss the challenges and controversies surrounding current efforts to advance them for potential use to prevent or treat COVID-19.

## PLANT-DERIVED MEDICINAL PRODUCTS FOR COVID-19 MANAGEMENT?

**Figure 1.**
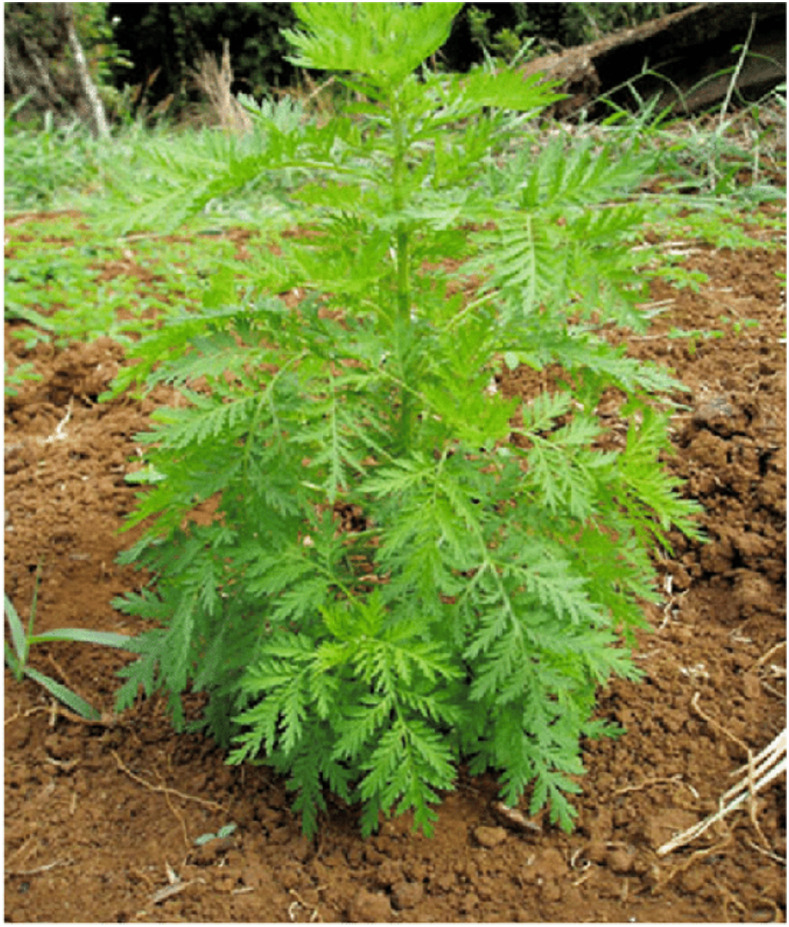
*Artemisia annua*.

**Figure 2.**
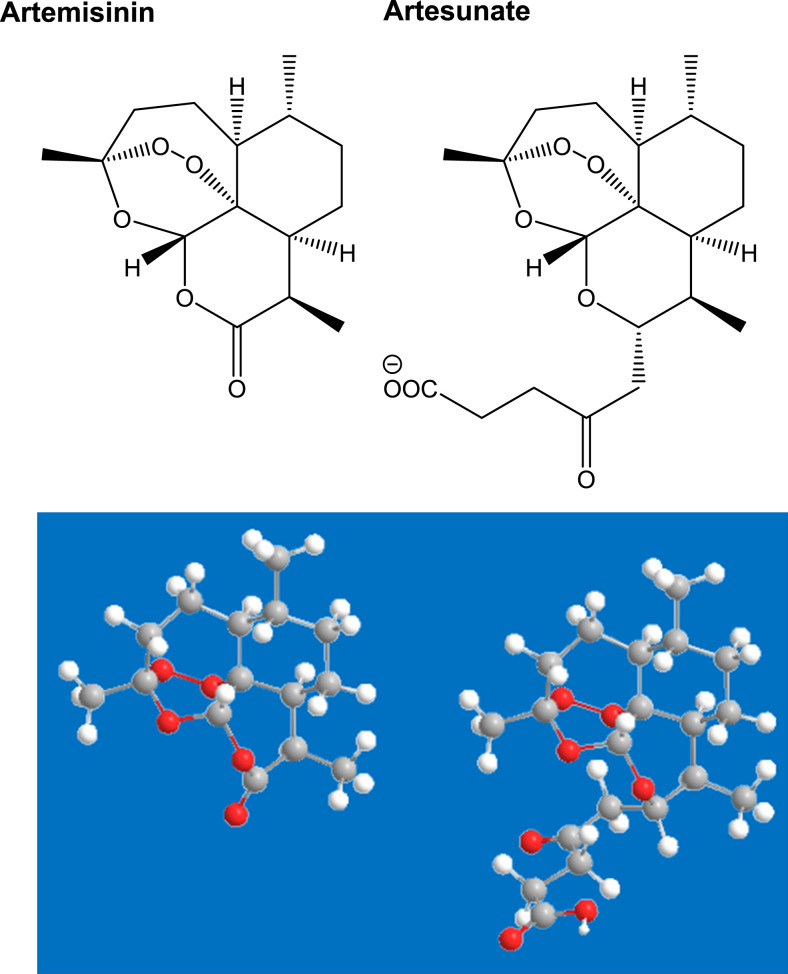
2D and 3D chemical structures of artemisinin and artesunate. In the 3D structure, carbon atoms appear as gray balls, hydrogen atoms as white balls, and oxygen atoms as red balls.

Traditional herb- and plant-derived medicinal products are being used and trialed for the treatment of COVID-19 in China.^6,7^ Among many, derivatives from the herb *Artemisia annua* (Figure 1) have been used as traditional medicine over centuries for the treatment of fevers, malaria, and respiratory tract infections.^8^ The “sweet wormwood” plant contains artemisinin (Figure 2), a medicine developed during the cultural revolution in China. The 2015 Nobel Prize in Physiology or Medicine was awarded to Professor Youyou Tu for her key contributions to its discovery.^9^ The WHO recommends artemisinin-based combination therapies (ACTs) as first-line treatments for uncomplicated *Plasmodium falciparum* malaria.^10^ Results of a small clinical study from China of artesunate, a hemi-synthetic derivative of artemisinin (Figure 2), for the treatment of COVID-19 reported that artesunate was associated with shorter duration of COVID-19 symptoms (3.3 ± 1.9 versus 4.8 ± 2.2 days) and hospital stays (16.6 ± 3.7 versus 18.0 ± 4.0) than standard of care.^8^ Table 1 shows the concentrations of artemisinin and artesunate that can be achieved in the plasma of patients receiving conventional doses of these drugs, and the concentrations needed to inhibit the replication of other viruses or the inflammatory response in vitro. These data suggest that artesunate may offer both antiviral and anti-inflammatory effects at clinically achievable concentrations.^8,11,12^

**Table 1:** Comparison of in vivo concentrations with in vitro active concentrations for artemisinin and artesunate^8–10^

Compound	Human doses	Human plasma concentrations (µg/L)	In vitro antiviral effects (IC_50_)	In vitro anti-inflammatory effects
Artemisinin	500 mg/day (po)	390–582	Flaviviruses	10–100 µM (2,820–28,200 µg/L)
			18.5 µM (5,217 µg/L)	
Artesunate	1–8 mg/kg/day (iv)	1,320–10,560	Herpesviruses	1–30 µM (384–11,520 µg/L)
			4–7 µM (1,536–2,688 µg/L)	
			HBV	
			10 µM (3,840 µg/L)	

IC_50_, 50% maximal inhibitory concentration, a measure which indicates how much a drug is needed to inhibit, in vitro, a given biological process by 50%.

In April 2020, an herbal tonic derived from *A. annua* extracts by the Madagascar Institute of Applied Research and branded “COVID-Organics” was launched. COVID-Organics has been promoted as a cure for COVID-19. However, reliable pharmacological and efficacy data are lacking, and there is concern that its widespread use for COVID-19 could result in reduced access to effective medicines as well as possible selection of *P. falciparum* resistance to ACTs by exposing patients to suboptimal concentrations of artemisinin when malaria cases are misdiagnosed as COVID-19. Of note, the content of artemisinin in *A. annua* is about 1%, which means that 50 g of plant material is needed to obtain the equivalent of a 500-mg therapeutic dose of artemisinin, if considering a 100% extraction.^13^ Furthermore, the typical concentration of artemisinin in infusions is around 50 mg/L; 10 L must be ingested to absorb the antimalarial therapeutic dose, which is not feasible. Therefore, to avoid the promotion of unproven remedies in this climate of uncertainty and fear, it is important that research into traditional medicinal plants and their derivatives be conducted properly.

## *ARTEMISIA ANNUA* AND BIOACTIVE COMPOUNDS

*Artemisia annua* is an annual herbaceous plant of the Asteraceae family native to Asia and Eastern Europe (Figure 1).^14^ As *A. annua* is the source for leading WHO-approved antimalarials, seed varieties have been adapted by breeding for lower latitudes, and cultivation has been successfully achieved in many tropical countries.^15^ *Artemisia annua* is a source of many biologically active compounds,^16,17^ with more than 220 compounds isolated and identified,^18^ including at least 28 monoterpenes, 30 sesquiterpenes, 12 triterpenoids and steroids, 36 flavonoids, seven coumarins, and four aromatic and nine aliphatic compounds.^15^ It naturally produces and stores artemisinin in the glandular trichomes on its leaves, stems, and flowers.^19^ Sesquiterpenes, caryophyllene oxide, caryophyllene, farnesene, and germacrene D are the most abundant chemicals identified in the essential oil of the fruits.^20^ Artemisinin is a sesquiterpene lactone, containing an unusual endoperoxide group (Figure 2) which is believed to be responsible for its antimalarial activity.

## *ARTEMISIA* SPP. PRODUCTS-ANTI-INFLAMMATORY AND IMMUNOMODULATORY EFFECTS AND COVID-19 TREATMENT POTENTIAL

*Artemisia annua* extracts are said to contain anti-inflammatory, antioxidant, and antimicrobial substances, and to show antiviral activity.^21–23^ The flavonoids casticin and chrysosplenol D, extracted from *A. annua*, suppressed the expression of inflammatory mediators through the regulation of NF-κB and c-JUN in a murine macrophage cell line, suggesting that these components might be useful in the treatment of inflammatory and infectious disorders.^24^ The water-soluble fraction of *A. annua*, after the extraction of artemisinin, was shown to regulate the expression of pro-inflammatory cytokines, matrix metalloproteinases, and NF-κB; promote cell cycle arrest; drive reactive oxygen species production; and induce Bak or Bax-dependent or independent apoptosis.^8^ *Artemisia annua* extracts significantly inhibited cytopathy caused by SARS-CoV strain BJ001^25^ and showed activity against SARS-CoV-2 in Vero-E6 cell-based cytopathic effect screening.^26^

## ANECDOTAL USE OF *A. ANNUA* PRODUCTS FOR COVID-19 TREATMENT

Since the beginning of the COVID-19 pandemic, formulations of *A. annua* have been used in Africa and China for COVID-19 prevention and treatment. In the DRC, herbal formulations have been used for the prevention and treatment of COVID-19 by fumigation, infusion, or decoction.^27^ It is important to emphasize that there are no controlled data supporting the use of any of these, and their efficacy for COVID-19 is unknown. Arguably, natural product research is only relevant to the development of new drugs as a first step to identifying specific molecules with activity. Teas cannot function as drugs meeting international standards, as their components are unknown and not standardized.^28^ Advancing traditional medicines will require identification of active components of plant extracts, methods to yield purified compounds, and determination of compound pharmacology including studies of biological activity, bioavailability, absorption, distribution, metabolism, excretion, and toxicity properties of each molecule.^28^ For evaluation of the antiviral effects of herbal formulations, the screening system should meet all requirements of any good assay, including validity, lack of ambiguity, accuracy, reproducibility, simplicity, and reasonable cost. Because these requirements are better met by in vitro screening, in vitro bioassays must be used to guide the isolation of active compounds from plant extracts. The antiviral activities of the pure compounds must then be confirmed at a later stage by in vivo assays in appropriate animal models.^23^

## ROAD MAP FOR *ARTEMISIA* SPP. DERIVATIVES AS A POTENTIAL THERAPEUTIC FOR COVID-19

The WHO acknowledges that the quantity and quality of safety and efficacy data on traditional medicines are far from sufficient to meet the criteria needed to support their use. The reasons for the lack of research data include inadequate healthcare policies and a lack of accepted research methodology for evaluating traditional medicines.^8,26,29^ At a time when countries are consumed by their own national interests and agendas, the world is looking to natural products to provide readily available, affordable treatments. A cure or treatment for COVID-19 derived from locally grown herbs and plants remains a viable option for some countries and communities. However, to develop drugs rather than crude preparations of herbs and plants, the specific pharmacologically active components need to be isolated, verified through proper pharmacological evaluation, and then possibly optimized through modern (hemi) synthesis strategies before being developed according to rigorous international guidelines for drug development. However, repurposing of available plant-based drugs, for example, artesunate, offers a potential time- and cost-saving approach. Indeed, investigators from Saudi Arabia have registered a placebo-controlled trial (www.ClinicalTrials.gov Identifier: NCT04387240) to evaluate the efficacy of artesunate in adults with mild symptoms of COVID-19.

## CONCLUSION

As the world desperately searches for new treatments to reduce rates of severe morbidity and mortality from COVID-19, the promotion of new drug discovery building on extracts from traditional medicinal plants should be encouraged. The anecdotal and unproven use of *A. Annua* for COVID-19 following claims from politicians and others in low-income countries highlights the need for hard data to establish the active ingredients; develop formulations and dosing; define the pharmacokinetics, toxicology, and safety; and evaluate efficacy through controlled trials.
