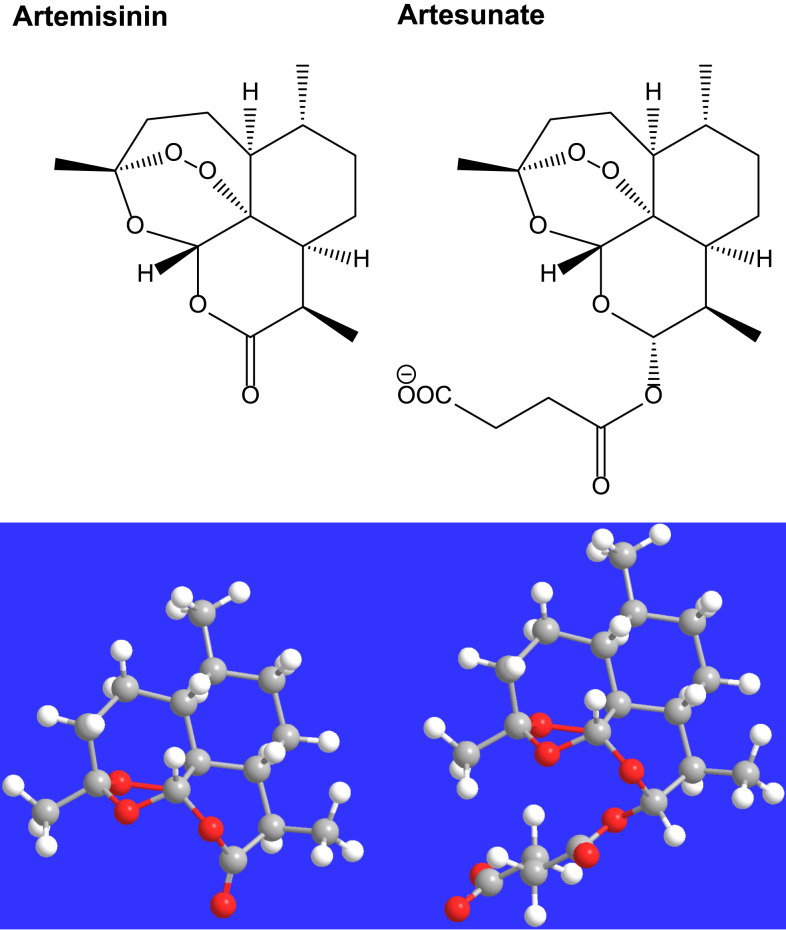# Erratum

**DOI:** 10.4269/ajtmh.20-0820err

**Published:** 2020-10-20

In “*Artemisia* Spp. Derivatives for COVID-19 Treatment: Anecdotal Use, Political Hype, Treatment Potential, Challenges, and Road Map to Randomized Clinical Trials” by Kapepula and others (https://www.ajtmh.org/content/journals/10.4269/ajtmh.20-0820) the structure of artesunate and the modelled structure are incorrect in Figure 2. The correct figure appears below.

**Figure 2.**